# Circulating osteoprotegerin as a cardiac biomarker for left ventricular diastolic dysfunction in patients with pre-dialysis chronic kidney disease: the KNOW-CKD study

**DOI:** 10.1007/s00392-024-02382-w

**Published:** 2024-02-06

**Authors:** Sang Heon Suh, Tae Ryom Oh, Hong Sang Choi, Chang Seong Kim, Eun Hui Bae, Seong Kwon Ma, Kook-Hwan Oh, Ji Yong Jung, Young Youl Hyun, Soo Wan Kim, the Korean Cohort Study for Outcomes in Patients With Chronic Kidney Disease (KNOW-CKD) Investigators

**Affiliations:** 1 Department of Internal Medicine, Chonnam National University Medical School and Chonnam National University Hospital, 42 Jebongro, Gwangju, 61469 Korea; 2 Department of Internal Medicine, Seoul National University Hospital, Seoul, Korea; 3 Division of Nephrology, Department of Internal Medicine, Gachon University Gil Medical Center, Incheon, Republic of Korea; 4 Department of Internal Medicine, Kangbuk Samsung Hospital, Sungkyunkwan University School of Medicine, Seoul, 03181 Republic of Korea

**Keywords:** Biomarker, Chronic kidney disease, Left ventricular diastolic dysfunction, Osteoprotegerin

## Abstract

**Background:**

Heart failure with preserved ejection fraction (HFpEF) is a major cause of mortality in patients with chronic kidney disease (CKD), and diagnosis is challenging. Moreover, no specific biomarker for HFpEF has been validated in patients with CKD. The present study aimed to investigate the association between serum osteoprotegerin (OPG) levels and the risk of left ventricular diastolic dysfunction (LVDD), a surrogate of HFpEF, in patients with pre-dialysis CKD.

**Methods:**

A total of 2039 patients with CKD at stage 1 to pre-dialysis 5 were categorized into quartiles (Q1 to Q4) by serum OPG levels, and were cross-sectionally analyzed. The study outcome was LVDD, which was operationally defined as the ratio of early transmitral blood flow velocity to early diastolic velocity of the mitral annulus (E/e’) > 14.

**Results:**

In the analysis of baseline characteristics, higher serum OPG levels were clearly related to the risk factors of HFpEF. A scatter plot analysis revealed a moderate correlation between serum OPG levels and E/e’ (*R* = 0.351, *P* < 0.001). Logistic regression analysis demonstrated that the risk of LVDD in Q3 (adjusted odds ratio 2.576, 95% confidence interval 1.279 to 5.188) and Q4 (adjusted odds ratio 3.536, 95% confidence interval 1.657 to 7.544) was significantly higher than that in Q1.

**Conclusions:**

Elevated serum OPG levels are associated with the risk of LVDD in patients with pre-dialysis CKD. The measurement of serum OPG levels may help the diagnosis of LVDD, which is an important echocardiographic feature of HFpEF.

**Graphical Abstract:**

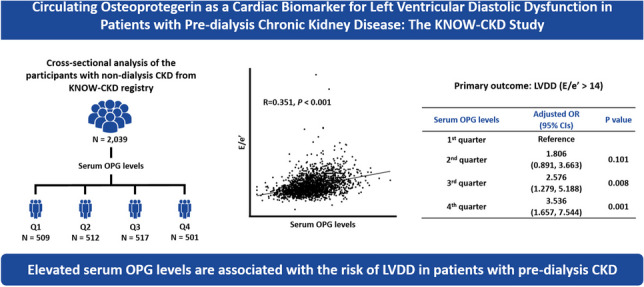

**Supplementary Information:**

The online version contains supplementary material available at 10.1007/s00392-024-02382-w.

## Introduction

Cardiovascular disease (CVD) is a leading cause of mortality in patients with chronic kidney disease (CKD) [1, 2]. Although coronary artery disease (CAD) is the most frequent CVD among the general population [3–5], the proportion of heart failure (HF) is relatively high in patients with CKD [6, 7]. According to the status of left ventricular ejection fraction (LVEF), HF is largely divided into HF with reduced ejection fraction (HFrEF) and HF with preserved ejection fraction (HFpEF) [8]. Current guidelines for HF therapeutics mainly focus on HFrEF [9–11], probably because high-quality evidence for HFpEF management has been available only recently [12, 13]. The impact of HFpEF has long been underestimated, and the mortality of HFpEF is almost as high as that of HFrEF in patients with CKD [14]. Moreover, HFpEF is an issue of particular importance, as it is far more frequent in patients with CKD than in the general population [15–17].

Left ventricular diastolic dysfunction (LVDD) is an echocardiographic feature of HFpEF [8, 9, 17] and is associated with adverse outcomes in patients with CKD. Not only systolic but also diastolic dysfunction of the left ventricle is associated with all-cause mortality in patients with end-stage renal disease (ESRD) [18]. LVDD is associated with the risk of both CKD progression [19, 20] and cardiovascular events [21] in patients with pre-dialysis CKD. Despite its substantial impact on CKD outcomes, echocardiographic examination is rarely performed largely owing to its limited accessibility [22]. The diagnosis of HFpEF is even more challenging because typical features of HF usually overlap with symptoms of volume retention from CKD itself [17]. In this regard, the use of biomarkers may help the diagnosis of HFpEF, although no specific biomarker has been validated in patients with CKD [17].

Osteoprotegerin (OPG) is a decoy receptor of activator of nuclear factor-κB ligand (RANKL) and a potent anti-osteoclastic molecule [23–26]. By blocking the binding between RANKL and receptor activator of nuclear factor-κB (RANK), OPG prevents excessive bone resorption, from which the name of OPG originated, “the bone protector” [23–26]. Yet, the clinical implications of circulating OPG are not limited to bone metabolism, as the association of OPG with cardiovascular outcomes has been suggested both in the general population and in patients with CKD [27, 28]. Specifically, the predictive role of serum OPG levels in CAD and coronary artery calcification has been repeatedly validated [29, 30], whereas the association between serum OPG levels and LVDD is still uncertain, especially among patients with pre-dialysis CKD.

The present study was designed to evaluate the association between serum OPG levels and the risk of LVDD in patients with pre-dialysis CKD. By analyzing the echocardiographic parameters from more than 2000 patients, we aimed to prove the hypothesis that elevation in serum OPG levels is associated with the risk of LVDD in patients with pre-dialysis CKD.

## Methods

### Study design

**Fig. 1:**
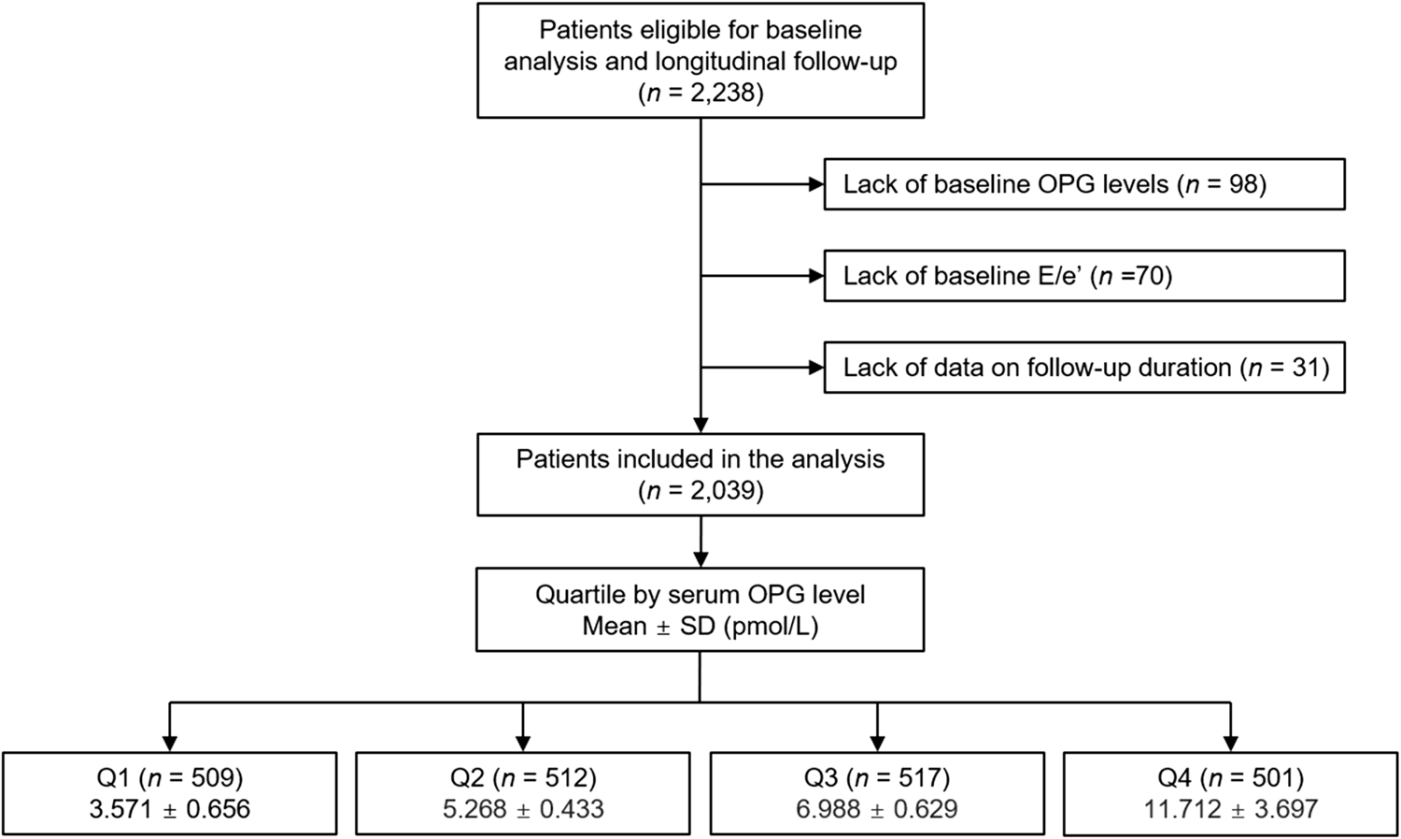
Flow diagram of the study participants. E/e’, ratio of early transmitral blood flow velocity to early diastolic velocity of the mitral annulus; OPG, osteoprotegerin; Q1, 1st quartile; Q2, 2nd quartile; Q3, 3rd quartile; Q4, 4th quartile; SD, standard deviation

The study protocol of the KNOW-CKD (KoreaN Cohort Study for Outcome in Patients With Chronic Kidney Disease) has been previously published [31]. Briefly, the patients with CKD at stage 1 to pre-dialysis 5 were initially recruited in South Korea from 2011 to 2016. According to the Declaration of Helsinki, all the participants voluntarily provided informed consent. Nine tertiary hospitals participated in the study, and the Institutional Review Board at each participating center reviewed and approved the study protocol. Among the 2238 participants initially enrolled, those lacking baseline serum OPG levels, baseline ratio of early transmitral blood flow velocity to early diastolic velocity of the mitral annulus (E/e’), or data on follow-up duration were excluded. Finally, a total of 2039 patients were included for further analyses (Fig. 1).

### Data collection

All the available data on demographics, anthropometrics, and laboratory measures were recorded at baseline according to the study protocol. Blood and urine samples were collected following overnight fasting and were transferred to the central laboratory for analyses (Lab Genomics, Seongnam, Korea). Estimated glomerular filtration rate (eGFR) was calculated using the Chronic Kidney Disease Epidemiology Collaboration equation with serum creatinine levels [32]. Spot urine samples were used to calculate the albumin-to-creatinine ratio (ACR). Echocardiographic examinations were conducted at each participating center according to a standard approach [33] by cardiologists blinded to the clinical data. Early diastolic mitral annulus velocity was measured by tissue Doppler in the septal region of the mitral annulus [19].

### Measurement of serum OPG levels

Serum OPG levels were measured using an enzyme-linked immunosorbent assay (ELISA) kit (BioVendor R&D, Brno, Czech Republic). The measurement was repeated twice, the mean of which was used in the current study. Three samples were below the detection range (< 1.5 pmol/L) and were approximated to 1.5 pmol/L.

### Exposure and study outcomes

The participants were categorized into quartiles (Q1, Q2, Q3, and Q4) by serum OPG levels (Fig. 1), the primary exposure of the present study. The study outcome was LVDD, which was operationally defined as E/e’ > 14 [21].

### Statistical analyses

One-way analysis of variance and χ^2^ test were used to analyze the baseline characteristics for continuous and categorical variables, respectively. To visualize the correlation between serum OPG levels and LVEF or E/e’, a scatter plot with Pearson’s correlation coefficient analysis was depicted. Instead of excluding all the participants who have any missing variables for the adjustment models in the binary logistic regression analyses, we first used the all the data available for one-way analysis of variance and χ^2^ test to analyze the baseline characteristics, and a scatter plot with Pearson’s correlation coefficient analysis. Binary logistic regression analyses were performed to demonstrate the independent association between serum OPG levels and the risk of LVDD. The adjustment models were as follows: Model 1 was unadjusted; Model 2 was adjusted for age and sex; Model 3 was additionally adjusted for Charlson comorbidity index, primary cause of CKD, smoking status, medication history (angiotensin-converting enzyme inhibitors and/or angiotensin II receptor blockers (ACEi/ARBs), diuretics, statins, and antiplatelets/anticoagulants), body mass index (BMI), and systolic blood pressure (SBP); and Model 4 was further adjusted for hemoglobin, albumin, high-density lipoprotein cholesterol (HDL-C), fasting glucose, high-sensitivity C-reactive protein (hs-CRP), 25-hydroxyvitamin D (25(OH)D), eGFR, and spot urine ACR. The subjects with any missing values for the adjustment models were excluded from the primary analysis. The results of logistic regression analyses are shown as odds ratio (OR) with 95% confidence interval (CI). Penalized spline curve analysis was performed to depict the linear correlation between serum OPG levels as a continuous variable and OR for LVDD. To validate our findings from the primary analyses, a series of sensitivity analyses were designed. First, the association between log-transformed serum OPG levels as a continuous variable and the risk of LVDD was evaluated by logistic regression analysis. Second, the participants were divided into tertiles or quintiles, instead of quartiles, by serum OPG levels, to repeat logistic regression analysis. Third, after replacing missing values using multiple imputation, logistic regression analysis was repeated. Fourth, we conducted a propensity score matching analysis of ORs for LVDD by serum OPG levels, where the patients were dichotomized (Q1 and Q2 *versus* (*vs.*) Q3 and Q4) and matched 1:1 by age, gender and eGFR using the nearest neighbor algorithm with a 0.1 caliper restriction (Table S4). We also conducted pre-specified subgroup analyses to address whether the association between serum OPG levels and the risk of LVDD was affected by certain clinical contexts. Subgroups were defined by age (< 60 *vs*. ≥ 60 years), sex (male *vs.* female), BMI (< 23 *vs.* ≥ 23 kg/m^2^), eGFR (< 45 *vs.* ≥ 45 mL/min/1.73 m^2^), and spot urine ACR (< 300 *vs.* ≥ 300 mg/g). Two-sided *P* values < 0.05 were considered statistically significant. Statistical analysis was performed using SPSS for Windows version 22.0 (IBM Corp., Armonk, NY) and R (version 4.1.1; R Project for Statistical Computing, Vienna, Austria).

## Results

### Baseline characteristics

The baseline characteristics of the participants significantly differed by serum OPG levels (Table 1). High serum OPG levels were related to older age, higher burden of comorbid conditions, higher prevalence of diabetes mellitus, higher proportion of current smoking, less intense treatment with diuretics, statins, and antiplatelets and/or anticoagulants, and higher blood pressure. Hemoglobin, albumin, HDL-C, 25(OH)D, and eGFR levels were lowest in the subjects with the highest serum OPG levels (Q4), whereas fasting glucose, hs-CRP, and spot urine ACR levels were highest in Q4. Echocardiographic findings also demonstrated a significant distinction by serum OPG levels (Table S1). Increased left ventricular mass index, left atrial diameter, posterior and inter-ventricular wall thickness, and left ventricular end-diastolic and end-systolic diameters were related to higher serum OPG levels. The frequencies of regional wall motion abnormality and valve calcification were also highest in Q4. Most importantly, E/e’ gradually increased with serum OPG levels. Taken together, elevation in serum OPG levels was clearly related to the risk factors of HFpEF at baseline.

**Table 1:** Baseline characteristics of study participants by serum OPG levels

	Serum OPG levels				*P* value
	Q1	Q2	Q3	Q4	
Age (year)	43.831 ± 10.924	51.332 ± 10.563	56.323 ± 10.479	62.711 ± 8.097	< 0.001
Male	330 (64.8)	293 (57.2)	305 (59.0)	311 (62.1)	0.064
Charlson comorbidity index					< 0.001
0–3	480 (94.3)	425 (83.0)	349 (67.5)	196 (39.1)	
4–5	29 (5.7)	84 (16.4)	153 (29.6)	287 (57.3)	
≥ 6	0 (0.0)	3 (0.6)	15 (2.9)	18 (3.6)	
Primary cause of CKD					< 0.001
DM	24 (4.7)	84 (16.4)	141 (27.3)	264 (52.7)	
HTN	88 (17.3)	101 (19.7)	123 (23.8)	86 (17.2)	
GN	240 (47.2)	190 (37.1)	145 (28.1)	70 (14.0)	
PKD	122 (24.0)	105 (20.5)	76 (14.7)	35 (7.0)	
Other	35 (6.9)	32 (6.3)	31 (6.0)	46 (9.2)	
Smoking status					0.045
Non-smoker	276 (54.2)	276 (53.9)	280 (54.3)	257 (51.3)	
Ex-smoker	95 (18.7)	90 (17.6)	64 (12.4)	78 (15.6)	
Current smoker	138 (27.1)	146 (28.5)	172 (33.3)	166 (33.1)	
Medication					
ACEi/ARBs	440 (86.4)	443 (86.5)	446 (86.3)	416 (83.0)	0.32
Diuretics	413 (81.1)	374 (73.0)	340 (65.8)	268 (53.5)	< 0.001
Statins	297 (58.3)	254 (49.6)	217 (42.0)	207 (41.3)	< 0.001
Antiplatelets/anticoagulants	423 (83.1)	379 (74.0)	357 (69.1)	308 (61.5)	< 0.001
BMI (kg/m^2^)	24.595 ± 3.598	24.622 ± 3.478	24.628 ± 3.446	24.408 ± 3.072	0.655
SBP (mmHg)	124.491 ± 14.751	126.588 ± 14.345	127.511 ± 15.533	132.900 ± 18.622	< 0.001
DBP (mmHg)	77.660 ± 10.911	78.092 ± 10.507	76.518 ± 10.738	75.788 ± 12.239	0.004
Laboratory findings					
Hemoglobin (g/dL)	13.798 ± 1.832	13.164 ± 1.922	12.704 ± 1.908	11.670 ± 1.781	< 0.001
Albumin (g/dL)	4.302 ± 0.349	4.228 ± 0.384	4.184 ± 0.398	3.990 ± 0.487	< 0.001
Total cholesterol (mg/dL)	175.690 ± 33.696	175.951 ± 39.936	174.184 ± 42.069	170.679 ± 40.490	0.127
LDL-C (mg/dL)	98.577 ± 28.697	98.585 ± 33.076	95.737 ± 33.144	93.898 ± 31.665	0.045
HDL-C (mg/dL)	50.627 ± 15.127	51.452 ± 15.847	48.468 ± 15.411	46.575 ± 14.929	< 0.001
TG (mg/dL)	155.088 ± 97.540	146.938 ± 82.574	165.737 ± 109.196	162.514 ± 100.614	0.007
Fasting glucose (mg/dL)	101.016 ± 21.924	105.217 ± 30.236	112.331 ± 38.477	124.120 ± 55.726	< 0.001
25(OH)D (ng/mL)	18.464 ± 7.334	18.207 ± 7.189	17.792 ± 7.266	17.000 ± 9.595	0.041
hs-CRP (mg/dL)	0.450 [0.200, 1.300]	0.700 [0.295, 1.500]	0.600 [0.300, 1.700]	0.700 [0.220, 2.100]	< 0.001
Spot urine ACR (mg/g)	209.262 [34.574, 601.352]	293.761 [50.457, 758.491]	389.824 [90.954, 1144.680]	711.006 [207.697, 2008.830]	< 0.001
Creatinine (mg/dL)	1.378 ± 0.973	1.571 ± 0.932	1.850 ± 1.027	2.485 ± 1.337	< 0.001
eGFR (mL/min./1.73 m^2^)	67.923 ± 32.239	56.886 ± 31.044	44.852 ± 23.939	31.648 ± 18.379	< 0.001
CKD stages					< 0.001
Stage 1	170 (33.4)	107 (20.9)	43 (8.3)	11 (2.2)	
Stage 2	142 (27.9)	125 (24.4)	88 (17.0)	29 (5.8)	
Stage 3a	84 (16.5)	84 (16.4)	100 (19.3)	63 (12.6)	
Stage 3b	67 (13.2)	105 (20.5)	144 (27.9)	118 (23.6)	
Stage 4	40 (7.9)	74 (14.5)	117 (22.6)	203 (40.5)	
Stage 5	6 (1.2)	17 (3.3)	25 (4.8)	77 (15.4)	

Values for categorical variables are given as number (percentage); values for continuous variables, as mean ± standard deviation or median [interquartile range]. *25(OH)D*, 25-hydroxyvitamin D; *ACEi/ARBs*, angiotensin-converting enzyme inhibitors and/or angiotensin receptor blockers; *ACR*, albumin-to-creatinine ratio; *BMI*, body mass index; *CACS*, coronary artery calcium score; *CKD*, chronic kidney disease; *DBP*, diastolic blood pressure; *DM*, diabetes mellitus; *eGFR*, estimated glomerular filtration rate; *GN*, glomerulonephritis; *HDL-C*, high-density lipoprotein cholesterol; *hs-CRP*, high-sensitivity C-reactive protein; *HTN*, hypertension; *LDL-C*, low-density lipoprotein cholesterol; *OPG*, osteoprotegerin; *PKD*, polycystic kidney disease; *Q1*, 1st quartile; *Q2*, 2nd quartile; *Q3*, 3rd quartile; *Q4*, 4th quartile; *SBP*, systolic blood pressure; *TG*, triglycerides

### Association between serum OPG levels and the risk of LVDD

**Fig. 2:**
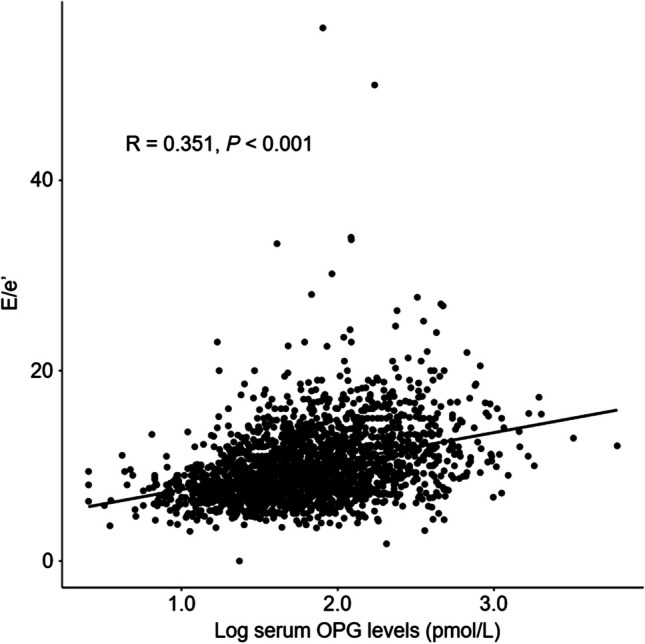
Scatter plot of serum OPG levels with E/e’. The correlation of serum OPG levels with E/e’ was assessed with the Pearson correlation coefficient (R). E/e’, ratio of early transmitral blood flow velocity to early diastolic velocity of the mitral annulus; OPG, osteoprotegerin

**Fig. 3:**
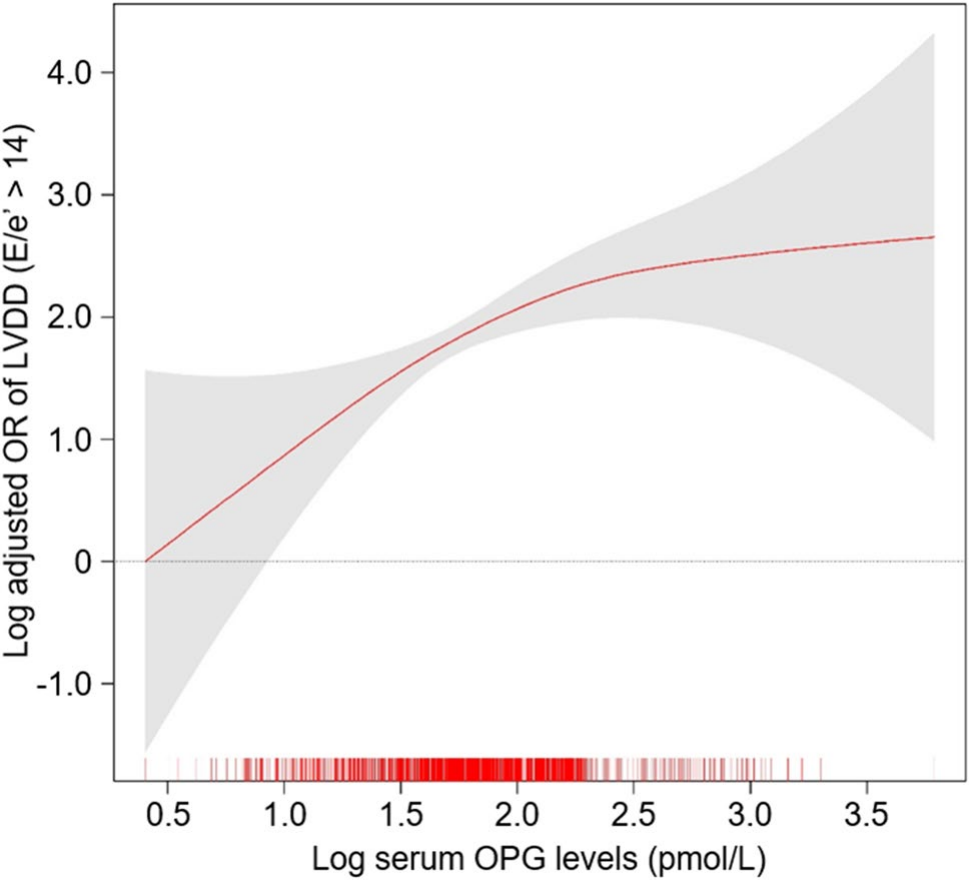
Penalized spline curve of serum OPG level on the risk of LVDD. Adjusted OR of serum OPG level as a continuous variable for the risk of LVDD is depicted. The model was adjusted for age, sex, Charlson comorbidity index, primary causes of CKD, smoking status, medication (ACEi/ARBs, diuretics, statins, and antiplatelets/anticoagulants), BMI, SBP, hemoglobin, albumin, HDL-C, fasting glucose, 25(OH)D, hs-CRP, eGFR, and spot urine ACR. E/e’, ratio of early transmitral blood flow velocity to early diastolic velocity of the mitral annulus; LVDD, left ventricular diastolic dysfunction; OR, odds ratio; OPG, osteoprotegerin

A scatter plot analysis revealed a moderate correlation between serum OPG levels and E/e’ (*R* = 0.351, *P* < 0.001) (Fig. 2), while no significant correlation between serum OPG levels and LVEF was observed (*R* = 0.001, *P* < 0.951) (Figure S1). In the binary logistic regression analysis, compared to that of Q1, ORs for LVDD in Q3 (adjusted OR 2.576, 95% CI 1.279 to 5.188) and Q4 (adjusted OR 3.536, 95% CI 1.657 to 7.544) were significantly increased (Table 2), suggesting an independent association between serum OPG levels and the risk of LVDD. Penalized spline curve analysis visualized a clear, linear association between serum OPG levels and the risk of LVDD (Fig. 3).

**Table 2:** ORs for LVDD by serum OPG levels

Outcome	Serum OPG levels (pmol/L)		Events, *n* (%)	Model 1		Model 2		Model 3		Model 4	
				OR (95% CI)	*P* value	OR (95% CI)	*P* value	OR (95% CI)	*P* value	OR (95% CI)	*P* value
LVDD (E/e’ > 14)	Q1	1.50–4.50	13 (2.6)	Reference		Reference		Reference		Reference	
	Q2	4.51–5.99	38 (7.4)	3.059 (1.609, 5.814)	< 0.001	2.470 (1.287, 4.743)	0.007	2.161 (1.107, 4.217)	0.024	1.806 (0.891, 3.663)	0.101
	Q3	6.01–8.26	68 (13.2)	5.778 (3.149, 10.601)	< 0.001	4.124 (2.189, 7.769)	< 0.001	3.249 (1.684, 6.266)	< 0.001	2.576 (1.279, 5.188)	0.008
	Q4	8.28–44.20	125 (25.0)	12.684 (7.055, 22.806)	< 0.001	7.820 (4.116, 14.856)	< 0.001	5.364 (2.678, 10.744)	< 0.001	3.536 (1.657, 7.544)	0.001

Model 1, unadjusted model. Model 2, model 1 + adjusted for age and sex. Model 3, model 2 + adjusted for Charlson comorbidity index, primary cause of CKD, smoking history, medication (ACEi/ARBs, diuretics, statins, and antiplatelets/anticoagulants), BMI, and SBP. Model 4, model 3 + adjusted for hemoglobin, albumin, HDL-C, fasting glucose, hs-CRP, 25(OH)D, eGFR, and spot urine ACR. CI, confidence interval; *E/e’*, ratio of early transmitral blood flow velocity to early diastolic velocity of the mitral annulus; *LVDD*, left ventricular diastolic dysfunction; *OPG*, osteoprotegerin; *OR*, odds ratio; *Q1*, 1st quartile; *Q2*, 2nd quartile; *Q3*, 3rd quartile; *Q4*, 4th quartile

### Sensitivity and subgroup analyses

Log-transformed serum OPG levels as a continuous variable were significantly associated with increased risk of LVDD (*per* 1 log increase, adjusted OR 2.077, 95% CI 1.251 to 3.448) (Table S2). After categorizing the participants into tertiles and quintiles, the risk of LVDD was significantly increased in the 3rd tertile (adjusted OR 1.843, 95% CI 1.029 to 3.301) and 5th quintile (adjusted OR 4.038, 95% CI 1.662 to 9.811), compared to the 1st tertile and 1st quintile, respectively (Table S3). After replacing missing values by multiple imputation, the risk of LVDD was still significantly increased in the subjects with higher serum OPG levels (Q2, adjusted OR 2.017, 95% CI 1.028 to 3.958; Q3, adjusted OR 2.713, 95% CI 1.390 to 5.296; Q4, adjusted OR 3.885, 95% CI 1.880 to 8.027), compared to Q1 (Table 3). Higher serum OPG levels were significantly associated with increased risk of LVDD (adjusted OR 1.925, 95% CI 1.167 to 3.718) even after the propensity score matching for age, gender, and eGFR (Table 4). Subgroup analyses revealed that the association between serum OPG levels and the risk of LVDD was not significantly modified by age, sex, BMI, eGFR, or albuminuria (all *P* for interactions > 0.05) (Table 5).

**Table 3:** ORs for LVDD by serum OPG levels after multiple imputation

Outcome	Serum OPG levels (pmol/L)	Model 1		Model 2		Model 3		Model 4	
		OR (95% CI)	*P* value	OR (95% CI)	*P* value	OR (95% CI)	*P* value	OR (95% CI)	*P* value
LVDD (E/e’ > 14)	Q1	Reference		Reference		Reference		Reference	
	Q2	3.059 (1.609, 5.814)	< 0.001	2.470 (1.287, 4.743)	0.007	2.169 (1.112, 4.229)	0.023	2.017 (1.028, 3.958)	0.041
	Q3	5.778 (3.149, 10.601)	< 0.001	4.124 (2.189, 7.769)	< 0.001	3.199 (1.659, 6.169)	< 0.001	2.713 (1.390, 5.296)	0.003
	Q4	12.684 (7.055, 22.086)	< 0.001	7.820 (4.116, 14.856)	< 0.001	5.246 (2.619, 10.509)	< 0.001	3.885 (1.880, 8.027)	< 0.001

Model 1, unadjusted model. Model 2, model 1 + adjusted for age and sex. Model 3, model 2 + adjusted for Charlson comorbidity index, primary cause of CKD, smoking status, medication (ACEi/ARBs, diuretics, statins, and antiplatelets/anticoagulants), BMI, and SBP. Model 4, model 3 + adjusted for hemoglobin, albumin, HDL-C, fasting glucose, hs-CRP, 25(OH)D, eGFR, and spot urine ACR. *CI*, confidence interval; *E/e’*, ratio of early transmitral blood flow velocity to early diastolic velocity of the mitral annulus; *LVDD*, left ventricular diastolic dysfunction; *OPG*, osteoprotegerin; *OR*, odds ratio; *Q1*, 1st quartile; *Q2*, 2nd quartile; *Q3*, 3rd quartile; *Q4*, 4th quartile

**Table 4:** Propensity score matching analysis of ORs for LVDD by serum OPG levels

Outcome	Serum OPG levels (pmol/L)		Events, *n* (%)	Model 1		Model 2		Model 3		Model 4	
				OR (95% CI)	*P* value	OR (95% CI)	*P* value	OR (95% CI)	*P* value	OR (95% CI)	*P* value
LVDD (E/e’ > 14)	Q1 + Q2	1.50–5.99	44 (5.1)	Reference		Reference		Reference		Reference	
	Q3 + Q4	6.01–44.20	136 (16.9)	2.634 (1.750, 3.964)	< 0.001	2.600 (1.724, 3.920)	< 0.001	2.017 (1.289, 3.157)	0.002	1.925 (1.167, 3.718)	0.010

Model 1, unadjusted model. Model 2, model 1 + adjusted for age and sex. Model 3, model 2 + adjusted for Charlson comorbidity index, primary cause of CKD, smoking status, medication (ACEi/ARBs, diuretics, statins, and antiplatelets/anticoagulants), BMI, and SBP. Model 4, model 3 + adjusted for hemoglobin, albumin, HDL-C, fasting glucose, hs-CRP, 25(OH)D, eGFR, and spot urine ACR. *CI*, confidence interval; *E/e’*, ratio of early transmitral blood flow velocity to early diastolic velocity of the mitral annulus; *LVDD*, left ventricular diastolic dysfunction; *OPG*, osteoprotegerin; *OR*, odds ratio; *Q1*, 1st quartile; *Q2*, 2nd quartile; *Q3*, 3rd quartile; *Q4*, 4th quartile

**Table 5:** ORs for LVDD by serum OPG levels in various subgroups

Outcome	Subgroups	Serum OPG levels (pmol/L)	Events, *n* (%)	Unadjusted OR (95% CI)	*P* for interaction	Adjusted OR (95% CI)	*P* for interaction
LVDD (E/e’ > 14)	Age < 60 years	1.50–4.50	12 (2.6)	Reference	0.149	Reference	0.403
		4.51–5.99	24 (5.9)	2.392 (1.203, 5.008)		1.463 (0.639, 3.499)	
		6.01–8.26	29 (9.8)	4.130 (2.123, 8.536)		1.691 (0.720, 4.145)	
		8.28–44.20	38 (24.1)	12.086 (6.303, 24.811)		2.980 (1.120, 8.239)	
	Age ≥ 60 years	1.50–4.50	1 (2.6)	Reference		Reference	
		4.51–5.99	14 (13.3)	5.846 (1.114, 107.796)		4.543 (0.813, 85.479)	
		6.01–8.26	39 (17.7)	8.188 (1.692, 147.490)		6.458 (1.267, 118.236)	
		8.28–44.20	87 (25.4)	12.914 (2.736, 230.930)		8.549 (1.667, 156.899)	
	Male	1.50–4.50	10 (3.0)	Reference	0.316	Reference	0.490
		4.51–5.99	18 (6.1)	1.444 (0.636, 3.279)		1.617 (0.692, 3.781)	
		6.01–8.26	40 (13.1)	2.407 (1.103, 5.251)		2.612 (1.155, 5.948)	
		8.28–44.20	67 (21.5)	3.475 (1.563, 7.728)		3.538 (1.411, 8.868)	
	Female	1.50–4.50	3 (1.7)	Reference		Reference	
		4.51–5.99	20 (9.1)	4.193 (1.183, 14.858)		3.594 (1.015, 12.728)	
		6.01–8.26	28 (13.2)	4.248 (1.193, 15.124)		3.806 (1.064, 13.616)	
		8.28–44.20	58 (30.5)	8.116 (2.272, 28.989)		6.048 (1.596, 22.913)	
	BMI < 25 kg/m^2^	1.50–4.50	2 (0.7)	Reference	0.095	Reference	0.164
		4.51–5.99	16 (5.5)	8.436 (2.372, 53.678)		5.243 (1.335, 35.534)	
		6.01–8.26	25 (8.4)	13.278 (3.908, 83.015)		6.140 (1.570, 41.726)	
		8.28–44.20	64 (21.4)	37.489 (12.198, 242.193)		10.988 (2.664, 77.070)	
	BMI ≥ 25 kg/m^2^	1.50–4.50	11 (5.2)	Reference		Reference	
		4.51–5.99	22 (10.0)	2.051 (0.988, 4.496)		1.250 (0.542, 3.033)	
		6.01–8.26	43 (20.0)	4.591 (2.375, 9.616)		2.051 (0.911, 4.938)	
		8.28–44.20	61 (30.7)	8.117 (4.280, 16.797)		2.211 (0.882, 5.875)	
	eGFR ≥ 45 mL/min/1.73 m^2^	1.50–4.50	5 (1.3)	Reference	0.058	Reference	0.091
		4.51–5.99	20 (6.6)	5.310 (2.121, 16.111)		3.102 (1.050, 11.463)	
		6.01–8.26	15 (7.2)	5.798 (2.210, 18.043)		3.210 (1.025, 12.327)	
		8.28–44.20	21 (21.9)	20.888 (8.219, 64.138)		7.032 (2.036, 29.076)	
	eGFR < 45 mL/min/1.73 m^2^	1.50–4.50	8 (6.1)	Reference		Reference	
		4.51–5.99	18 (8.5)	1.434 (0.624, 3.587)		1.045 (0.416, 2.810)	
		6.01–8.26	53 (17.2)	3.183 (1.549, 7.428)		1.873 (0.808, 4.803)	
		8.28–44.20	104 (25.7)	5.312 (2.665, 12.155)		2.275 (0.914, 6.205)	
	Spot urine ACR < 300 mg/g	1.50–4.50	6 (2.0)	Reference	0.410	Reference	0.759
		4.51–5.99	17 (6.7)	3.429 (1.400, 9.628)		2.036 (0.754, 6.145)	
		6.01–8.26	25 (11.0)	5.911 (2.538, 16.148)		2.302 (0.827, 7.180)	
		8.28–44.20	24 (15.4)	8.727 (3.707, 24.013)		2.647 (0.841, 9.231)	
	Spot urine ACR ≥ 300 mg/g	1.50–4.50	7 (3.3)	Reference		Reference	
		4.51–5.99	19 (7.7)	2.415 (1.038, 6.292)		1.458 (0.563, 4.273)	
		6.01–8.26	43 (15.4)	5.236 (2.451, 12.965)		2.506 (1.006, 7.208)	
		8.28–44.20	98 (29.4)	12.034 (5.858, 29.091)		3.797 (1.432, 11.504)	

Adjusted OR of serum OPG level as a continuous variable for the risk of LVDD is depicted. The model was adjusted for age, sex, Charlson comorbidity index, primary causes of CKD, smoking status, medication (ACEi/ARBs, diuretics, statins, antiplatelets/anticoagulants), BMI, SBP, hemoglobin, albumin, HDL-C, fasting glucose, 25(OH)D, hs-CRP, eGFR, and spot urine ACR. *BMI*, body mass index; *eGFR*, estimated glomerular filtration rate; *ACR*, albumin-to-creatinine ratio; *OR*, odds ratio; *OPG*, osteoprotegerin

## Discussion

In the present study, we found that elevation in serum OPG levels is associated with the risk of LVDD in patients with pre-dialysis CKD. Serum OPG levels correlated well with E/e’, a surrogate of LVDD, but not with LVEF, a surrogate of systolic function, and were linearly associated with the risk of LVDD even after adjustment for potential confounding factors (Graphical abstract). The association was consistently observed regardless of clinical contexts, such as age, sex, BMI, eGFR, or albuminuria.

Although insufficient for the diagnosis of HFpEF, LVDD is an echocardiographic feature of HFpEF. In comparison to HFrEF, the diagnosis of which is mainly guided by a single echocardiographic index, the establishment of HFpEF is more complicated. The rigorous determination LVDD per se requires the fulfillment of a diagnostic criteria [34]. Therefore, in this regard, we assumed that the use of a biomarker may help the identification of LVDD, which is one of the important echocardiographic features of HFpEF.

The relation between serum OPG levels and LVDD has been previously suggested in the general population, as the study reported a significant correlation of serum OPG levels with left ventricular mass index and the other parameters of myocardial stiffness [35]. The mean eGFR of the subjects in the study was, however, approximately 89 mL/min/1.73 m^2^, because the study was based on a cohort of community-dwelling African-Americans with essential hypertension [35], suggesting that most of the subjects should have nearly normal kidney function. Although a previous study suggested the probable association between serum OPG levels and LVDD, the number of subjects was relatively small (*n* = 101), and the adjustment for confounding factors was not stringent [36]. To the best of our knowledge, the current study is the first large-scale study to demonstrate the association between serum OPG levels and the risk of LVDD in patients with pre-dialysis CKD.

Although the mortality of patients with HFpEF is lower than of those with HFrEF in the general population [37, 38], this is not the case in patients with CKD, as all-cause mortality in HFpEF is as high as in HFrEF in these patients [14]. Moreover, the prevalence of HFpEF is higher in patients with CKD than in the general population, accounting for about 50% of HF cases [15, 17]. In this regard, it is not surprising that CKD and HFpEF share many risk factors, including hypertension, diabetes, advanced age, and concurrent cardiovascular diseases [17]. Mechanistically, it is postulated that these conditions all contribute to cardiac remodeling, such as concentric hypertrophy (left ventricular hypertrophy) and cardiac fibrosis, subsequently leading to relaxation abnormality of the left ventricle [39]. LVDD is an echocardiographic feature in HFpEF, and is determined by a combination of the following echocardiographic parameters: mitral flow velocity, E/e’, peak velocity of tricuspid regurgitation jet, and left atrial maximum volume index [40]. Among these, E/e’ represents an indirect assessment of mean pulmonary capillary wedge pressure (or left ventricular filling pressure) and correlates with fibrosis of the left ventricle [40].

The precise mechanism underlying the association between serum OPG levels and the risk of LVDD remains to be elucidated. Yet, the experimental evidence so far suggests that the biological role of circulating OPG may be compensatory to the development of HF. Indeed, infusion of angiotensin II in mice results in cardiac hypertrophy, which is functionally decompensated in OPG knock-out mice [41]. In a murine model of age-related cardiac hypertrophy, the phenotype is much more severe in OPG-deficient mice [42]. OPG deletion results in aortic rupture and/or dissection in an angiotensin II-induced hypertensive mouse model [43]. These results collectively indicate that OPG promotes cardiovascular protection. Accordingly, circulating OPG seems more likely to be a cardiac biomarker, rather than a mediator, of LVDD.

N-terminal pro-brain natriuretic peptide (NT-proBNP) is the best cardiac biomarker related to the diagnosis and prognosis of HF in the general population [44]. Its use for the diagnosis of HFrEF has been also validated in patients with pre-dialysis CKD [45, 46], although a higher diagnostic threshold value is needed. In contrast, its diagnostic accuracy for HFpEF is less evident [17]. Contrary to the report that serum NT-proBNP levels correlate with left ventricular mass in patients with CKD [47], another study reported that NT-proBNP is not significantly associated either with left ventricular mass index, left atrial volume index, or LVDD. Interestingly, in the present study, the analysis of baseline characteristics (Table 1) showed that elevation of serum OPG levels was significantly associated with advanced age, uncontrolled blood pressure, higher prevalence of diabetes, and higher burden with comorbid conditions, all of which are risk factors for HFpEF. Moreover, echocardiographic findings revealed that serum OPG levels were also positively correlated with left ventricular mass index and left atrium diameter, but not with left ventricular ejection fraction (Table S1). Thus, our findings suggest a novel role of circulating OPG as a cardiac biomarker for HFpEF in patients with pre-dialysis CKD.

The present study has several limitations. First, owing to the cross-sectional design, we could not confirm the causal relation between serum OPG levels and the risk of LVDD, despite the biological evidence that circulating OPG seems more likely to be a cardiac biomarker rather than a mediator of LVDD [41–43]. Second, as all the data, including serum OPG levels and E/e’, were obtained once at baseline, the current study cannot determine whether serum OPG levels may increase or decrease in conjunction with LVDD status. Third, the results are based on a cohort of ethnically Korean subjects residing in South Korea, and, therefore, extrapolation of the current findings to other populations requires caution.

In conclusion, we report that elevation in serum OPG levels was associated with the risk of LVDD in patients with pre-dialysis CKD. It is expected that the measurement of serum OPG levels may help the diagnosis of HFpEF in this population.

## Supplementary Information

Below is the link to the electronic supplementary material.

Supplementary file1 (DOCX 209 KB)
