# Supplementary material for: Circulating osteoprotegerin as a cardiac biomarker for left ventricular diastolic dysfunction in patients with pre-dialysis chronic kidney disease: the KNOW-CKD study
Source: Clin Res Cardiol. 2024 Feb 6;113(11):1555–64. doi: 10.1007/s00392-024-02382-w (PMC11493791; doi:10.1007/s00392-024-02382-w)
Supplement: Supplementary file 1 — Supplementary file1 (DOCX 209 KB) [file 392_2024_2382_MOESM1_ESM.docx]

**- Supplementary Information -**

**Circulating osteoprotegerin as a cardiac biomarker for left ventricular diastolic dysfunction in patients with pre-dialysis chronic kidney disease: The KNOW-CKD study**

Sang Heon Suh, M.D., Ph.D.^1^, Tae Ryom Oh, M.D., Ph.D.^1^, Hong Sang Choi, M.D., Ph.D.^1^, Chang Seong Kim, M.D., Ph.D.^1^, Eun Hui Bae, M.D., Ph.D.^1^, Seong Kwon Ma, M.D., Ph.D.^1^, Kook-Hwan Oh, M.D., Ph.D.^2^, Ji Yong Jung, M.D., Ph.D.^3^, Young Youl Hyun, M.D., Ph.D.^4^, and Soo Wan Kim*, M.D., Ph.D.^1^, on behalf of the Korean Cohort Study for Outcomes in Patients With Chronic Kidney Disease (KNOW-CKD) Investigators

^1^Department of Internal Medicine, Chonnam National University Medical School and Chonnam National University Hospital, Gwangju, Korea

^2^Department of Internal Medicine, Seoul National University Hospital, Seoul, Korea

^3^Division of Nephrology, Department of Internal Medicine, Gachon University Gil Medical Center, Incheon, Republic of Korea

^4^Department of Internal Medicine, Kangbuk Samsung Hospital, Sungkyunkwan University School of Medicine, Seoul 03181, Republic of Korea

**Running title:** OPG and LVDD in CKD

**Corresponding authors**

*Soo Wan Kim, M.D., Ph.D., Department of Internal Medicine, Chonnam National University Medical School, 42 Jebongro, Gwangju 61469, Korea, Tel: +82-62-220-6271, Fax: +82-62-225-8578, Email: skimw@chonnam.ac.kr

**Table of Contents**

Figure S1. Scatter plot of serum OPG levels with LVEF

Table S1. Echocardiographic findings of study participants by serum OPG levels

Table S2. ORs for LVDD by log-transformed serum OPG levels as a continuous variable

Table S3. ORs for LVDD by serum OPG levels divided into tertiles and quintiles

Table S4. Baseline characteristics of study participants by serum OPG levels after propensity score matching of age, gender and eGFR


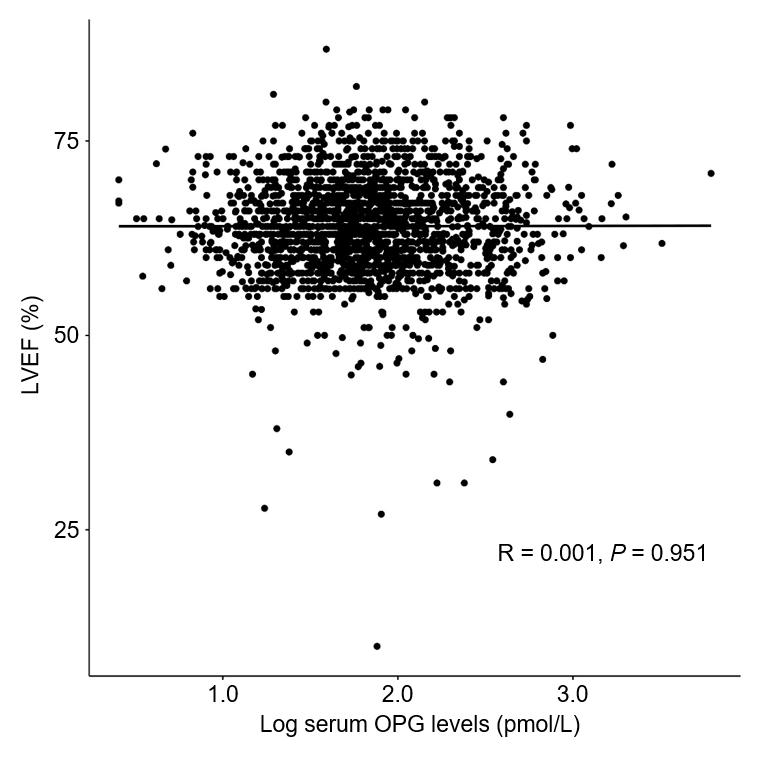


**Figure S1. Scatter plot of serum OPG levels with LVEF**

The correlation of serum OPG levels with E/e’ was assessed with the Pearson correlation coefficient (R). LVEF, left ventricular ejection fraction; OPG, osteoprotegerin.

**Table S1. Echocardiographic findings of study participants by serum OPG levels**

|  | Serum OPG levels | | | |  |
| --- | --- | --- | --- | --- | --- |
|  | Q1 | Q2 | Q3 | Q4 | *P* value |
| E/e’ | 8.282 ± 2.627 | 9.338 ± 3.160 | 10.328 ± 4.325 | 11.852 ± 4.354 | < 0.001 |
| LVMI (g/m^2^) | 86.107 ± 21.308 | 89.858 ± 21.329 | 95.534 ± 26.290 | 102.325 ± 26.602 | < 0.001 |
| LVEF (%) | 63.727 ± 5.706 | 64.485 ± 5.837 | 64.108 ± 6.599 | 63.876 ± 6.777 | 0.184 |
| LAD (mm) | 36.383 ± 5.341 | 36.961 ± 5.590 | 38.291 ± 5.722 | 39.215 ± 5.865 | < 0.001 |
| RWMA | 4 (0.8) | 8 (1.6) | 19 (3.7) | 33 (6.6) | < 0.001 |
| Valve calcification | 9 (1.8) | 27 (5.3) | 57 (11.0) | 90 (18.0) | < 0.001 |
| PWT (mm) | 8.851 ± 1.510 | 9.015 ± 1.430 | 9.391 ± 1.601 | 9.795 ± 1.609 | < 0.001 |
| IVWT (mm) | 8.872 ± 1.552 | 9.190 ± 1.540 | 9.528 ± 1.765 | 9.851 ± 1.829 | < 0.001 |
| LVEDD (mm) | 48.883 ± 4.116 | 48.456 ± 4.129 | 48.538 ± 4.841 | 48.720 ± 4.785 | 0.371 |
| LVESD (mm) | 30.806 ± 3.704 | 29.951 ± 3.753 | 30.271 ± 4.560 | 30.493 ± 4.933 | 0.003 |

Values for categorical variables are given as number (percentage); values for continuous variables, as mean ± standard deviation. E/e’, ratio of early transmitral blood flow velocity to early diastolic velocity of the mitral annulus; IVWT, interventricular wall thickness; LAD, left atrium diameter; LVEDD, left ventricular end-diastolic diameter; LVEF, left ventricular ejection fraction; LVESD, left ventricular end-systolic diameter; LVMI, left ventricular mass index; PWT, posterior wall thickness; Q1, 1^st^ quartile; Q2, 2^nd^ quartile; Q3, 3^rd^ quartile; Q4, 4^th^ quartile; RMWA, regional wall motion abnormality.

**Table S2. ORs for LVDD by log-transformed serum OPG levels as a continuous variable**

| Outcome | Serum OPG levels | Model 1 | | Model 2 | | Model 3 | | Model 4 | |
| --- | --- | --- | --- | --- | --- | --- | --- | --- | --- |
|  |  | OR  (95%CI) | *P* value | OR  (95%CI) | *P* value | OR  (95%CI) | *P* value | OR  (95%CI) | *P* value |
| LVDD  (E/e’ > 14) | *Per* 1 log increase | 5.718  (4.193, 7.798) | < 0.001 | 4.086  (2.827, 5.906) | < 0.001 | 2.862  (1.862, 4.399) | < 0.001 | 2.077  (1.251, 3.448) | 0.005 |

Model 1, unadjusted model. Model 2, model 1 + adjusted for age and sex. Model 3, model 2 + adjusted for Charlson comorbidity index, primary cause of CKD, smoking status, medication (ACEi/ARBs, diuretics, statins, and antiplatelets/anticoagulants), BMI, and SBP. Model 4, model 3 + adjusted for hemoglobin, albumin, HDL-C, fasting glucose, hs-CRP, 25(OH)D, eGFR, and spot urine ACR. CI, confidence interval; E/e’, ratio of early transmitral blood flow velocity to early diastolic velocity of mitral annulus; OPG, osteoprotegerin; OR, odds ratio.

**Table S3. ORs for LVDD by serum OPG levels divided into tertiles and quintiles**

| Outcome | Serum OPG levels (pmol/L) | | Events, n (%) | Model 1 | | Model 2 | | Model 3 | | Model 4 | |
| --- | --- | --- | --- | --- | --- | --- | --- | --- | --- | --- | --- |
|  |  |  |  | OR  (95%CI) | *P* value | OR  (95%CI) | *P* value | OR  (95%CI) | *P* value | OR  (95%CI) | *P* value |
| LVDD  (E/e’ > 14) | 1^st^ tertile | 1.50 – 5.00 | 27 (4.0) | Reference |  | Reference |  | Reference |  | Reference |  |
|  | 2^nd^ tertile | 5.01 – 7.29 | 65 (9.5) | 2.524  (1.590, 4.006) | < 0.001 | 1.872  (1.157, 3.029) | 0.011 | 1.501  (0.911, 2.476) | 0.111 | 1.231  (0.724, 2.092) | 0.444 |
|  | 3^rd^ tertile | 7.30 – 44.20 | 152 (22.4) | 6.933  (4.532, 10.605) | < 0.001 | 4.177  (2.585, 6.749) | < 0.001 | 2.746  (1.621, 4.653) | < 0.001 | 1.843  (1.029, 3.301) | 0.040 |
|  | 1^st^ quintile | 1.50 – 4.17 | 8 (2.0) | Reference |  | Reference |  | Reference |  | Reference |  |
|  | 2^nd^ quintile | 4.18 – 5.43 | 32 (7.8) | 4.223  (1.921, 9.281) | < 0.001 | 3.463  (1.565, 7.665) | 0.002 | 3.542  (1.580, 7.938) | 0.002 | 2.781  (1.213, 6.376) | 0.016 |
|  | 3^rd^ quintile | 5.44 – 6.71 | 36 (8.8) | 4.815  (2.209, 10.492) | < 0.001 | 3.489  (1.572, 7.743) | 0.002 | 2.720  (1.202, 6.156) | 0.016 | 2.116  (0.914, 4.894) | 0.080 |
|  | 4^th^ quintile | 6.72 – 8.95 | 60 (14.6) | 8.529  (4.022, 18.083) | < 0.001 | 5.600  (2.561, 12.244) | < 0.001 | 4.375  (1.948, 9.823) | < 0.001 | 2.891  (1.246, 6.705) | 0.013 |
|  | 5^th^ quintile | 8.97 – 44.20 | 108 (26.6) | 18.030  (8.657, 37.551) | < 0.001 | 10.439  (4.751, 22.937) | < 0.001 | 6.790  (2.945, 15.652) | < 0.001 | 4.038  (1.662, 9.811) | 0.002 |

Model 1, unadjusted model. Model 2, model 1 + adjusted for age and sex. Model 3, model 2 + adjusted for Charlson comorbidity index, primary cause of CKD, smoking status, medication (ACEi/ARBs, diuretics, statins, and antiplatelets/anticoagulants), BMI, and SBP. Model 4, model 3 + adjusted for hemoglobin, albumin, HDL-C, fasting glucose, hs-CRP, 25(OH)D, eGFR, and spot urine ACR. CI, confidence interval; E/e’, ratio of early transmitral blood flow velocity to early diastolic velocity of the mitral annulus; LVDD, left ventricular diastolic dysfunction; OPG, osteoprotegerin; OR, odds ratio.

**Table S4. Baseline characteristics of study participants by serum OPG levels after propensity score matching of age, gender and eGFR**

|  | Serum OPG levels | |  |
| --- | --- | --- | --- |
|  | Q1 + Q2  (*n* = 570) | Q3 + Q4  (*n* = 570) | *P* value |
| Follow-up duration (year) | 7.49 ± 2.65 | 7.04 ± 2.96 | 0.005 |
| Age (year) | 52.98 ± 10.20 | 53.44 ± 9.92 | 0.437 |
| Male | 229 (40.2) | 223 (39.1) | 0.762 |
| Charlson comorbidity index |  |  | <0.001 |
| 0 – 3 | 372 (65.3) | 267 (46.8) |  |
| 4 – 5 | 155 (27.2) | 227 (39.8) |  |
| ≥ 6 | 43 (7.5) | 76 (13.3) |  |
| Primary cause of CKD |  |  | N/A |
| DM | 74 (13.0) | 190 (33.5) |  |
| HTN | 131 (23.0) | 99 (17.4) |  |
| GN | 228 (40.0) | 152 (26.8) |  |
| PKD | 87 (15.3) | 86 (15.1) |  |
| Others | 50 (8.7) | 41 (7.2) |  |
| Smoking status |  |  | 0.252 |
| Non-smoker | 306 (53.7) | 298 (52.5) |  |
| Ex-smoker | 86 (15.1) | 106 (18.7) |  |
| Current smoker | 178 (31.2) | 164 (28.9) |  |
| Medication |  |  |  |
| ACEi/ARBs | 501 (87.9) | 497 (87.3) | 0.848 |
| Diuretics | 155 (27.2) | 176 (30.9) | 0.185 |
| Statins | 298 (52.3) | 304 (53.4) | 0.743 |
| Antiplatelets/anticoagulants | 143 (25.1) | 157 (27.6) | 0.372 |
| BMI (kg/m^2^) | 24.68 ± 3.22 | 24.43 ± 3.36 | 0.205 |
| SBP (mmHg) | 125.02 ± 14.78 | 129.43 ± 16.97 | <0.001 |
| DBP (mmHg) | 77.25 ± 10.69 | 77.76 ± 11.31 | 0.428 |
| Laboratory findings |  |  |  |
| Hemoglobin (g/dL) | 13.19 ± 1.90 | 12.66 ± 1.96 | <0.001 |
| Albumin (g/dL) | 4.25 ± 0.34 | 4.11 ± 0.48 | <0.001 |
| Total cholesterol (mg/dL) | 172.93 ± 35.75 | 177.26 ± 42.87 | 0.066 |
| LDL-C (mg/dL) | 96.27 ± 30.15 | 98.07 ± 34.82 | 0.354 |
| HDL-C (mg/dL) | 49.91 ± 14.88 | 49.49 ± 15.61 | 0.646 |
| TG (mg/dL) | 150.90 ± 85.21 | 165.69 ± 115.64 | 0.016 |
| Fasting glucose (mg/dL) | 105.07 ± 29.61 | 117.75 ± 51.27 | <0.001 |
| 25(OH)D (ng/mL) | 18.91 ± 7.52 | 17.07 ± 7.51 | <0.001 |
| hs-CRP (mg/dL) | 1.77 ± 3.73 | 2.01 ± 4.84 | 0.368 |
| Spot urine ACR (mg/g) | 605.53 ± 886.81 | 1126.00 ± 1618.92 | <0.001 |
| Creatinine (mg/dL) | 1.69 ± 1.07 | 1.79 ± 1.10 | 0.119 |
| eGFR (mL/min./1.73m^2^) | 50.84 ± 26.46 | 48.61 ± 25.88 | 0.151 |
| CKD stages |  |  | 0.400 |
| Stage 1 | 64 (11.2) | 63 (11.1) |  |
| Stage 2 | 146 (25.6) | 119 (20.9) |  |
| Stage 3a | 116 (20.4) | 123 (21.6) |  |
| Stage 3b | 132 (23.2) | 130 (22.8) |  |
| Stage 4 | 91 (16.0) | 109 (19.1) |  |
| Stage 5 | 21 (3.7) | 26 (4.6) |  |

Note: Values for categorical variables are given as number (percentage); values for continuous variables, as mean ± standard deviation or median [interquartile range]. Abbreviations: 25(OH)D, 25-hydroxyvitamin D; ACEi/ARBs, angiotensin converting enzyme inhibitors and/or angiotensin receptor blockers; ACR, albumin-to-creatinine ratio; BMI, body mass index; CACS, coronary artery calcium score; CKD, chronic kidney disease; DBP, diastolic blood pressure; DM, diabetes mellitus; eGFR, estimated glomerular filtration rate; GN, glomerulonephritis; HDL-C, high density lipoprotein cholesterol; hs-CRP, high-sensitivity C-reactive protein; HTN, hypertension; LDL-C, low density lipoprotein cholesterol; OPG, osteoprotegerin; PKD, polycystic kidney disease; Q1, 1st quartile; Q2, 2nd quartile; Q3, 3rd quartile; Q4, 4th quartile; SBP, systolic blood pressure; TG, triglycerides.
